# Supplementary material for: Automated detection of complex zebrafish seizure behavior at scale
Source: Commun Biol. 2025 Jun 5;8:872. doi: 10.1038/s42003-025-08310-6 (PMC12141442; doi:10.1038/s42003-025-08310-6)
Supplement: Supplementary file 1 — Supplementary Material [file 42003_2025_8310_MOESM1_ESM.pdf]

Supplementary information

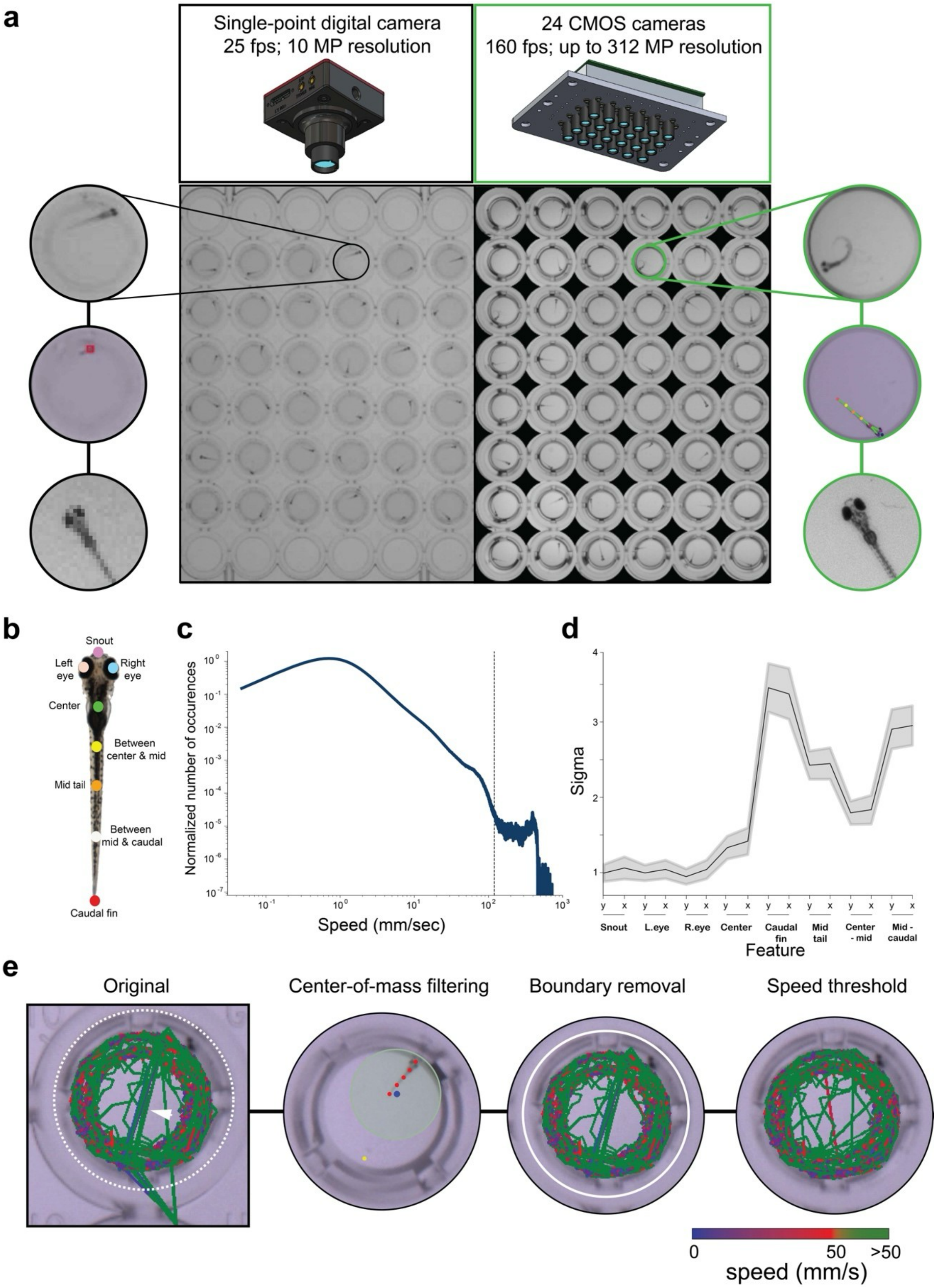

Supplemental Figure 1: Multicamera Array Microscope (MCAM). a. Specifications of a traditional single-camera tracking system compared to MCAM system. Representative images are shown to show sample resolution when recording in a high frame rate mode (6 MP at 160 fps) (top), tracking sample with single and multi-points (middle), resolution sample when recording in high resolution mode (312 MP at 15 fps) (bottom). **b.** Schematic showing the skeletal 8 key-points with labels used for tracking. **c.** Plot of all speed values on a log scale across all frames of the dataset totaling approximately 143 million data points to calculate maximum possible biological speed reached by zebrafish larvae. Dashed line indicates the calculated cutoff speed of 120 mm/s. **d.** The mean sigma, estimate of noise, plotted for 1,632 larvae for each key point feature. Gray shading represents standard error of the mean (SEM). Sigma values are used as an input to the wavelet denoising algorithm used to smooth data. Images shown in **e** from left to right represent calculated components that contribute to final tracking outputs. In the raw tracking plot, the dotted line indicates boundary of the well (original). White arrowhead indicates a sample of non-biological tracks. A center-of-mass (COM) is computed as the average of 6 tracking points along the larvae (snout, center and 4 tail points - points in red and yellow). Any point greater than 0.7x the fish length from the COM (green shaded region) is excluded (point in yellow). Tracking points that are captured beyond the well boundary are then removed (boundary removal). The speed cutoff is also applied to remove non biological tracking and a threshold is applied for visualization purposes (speed threshold). Blue (slow) to red (fast) color scale for speeds between 0 and 50 mm/s; green tracks represent swim speeds over 50 mm/s.

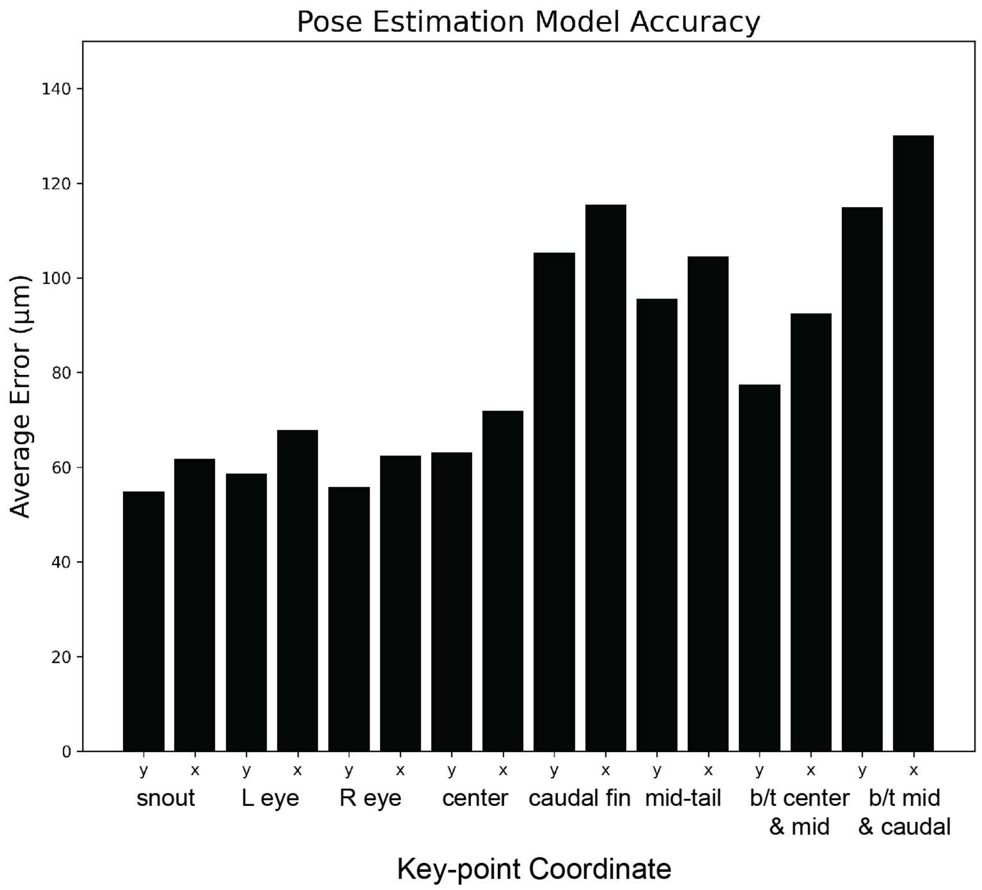

Supplemental Figure 2: Evaluation of the pose estimation model. 1,177 images of zebrafish in 96-well plate wells were manually annotated with eight key points and used as ground truth for comparison to be inferred key point locations. Average distance between inference and ground truth for each component of each key point is given in microns.

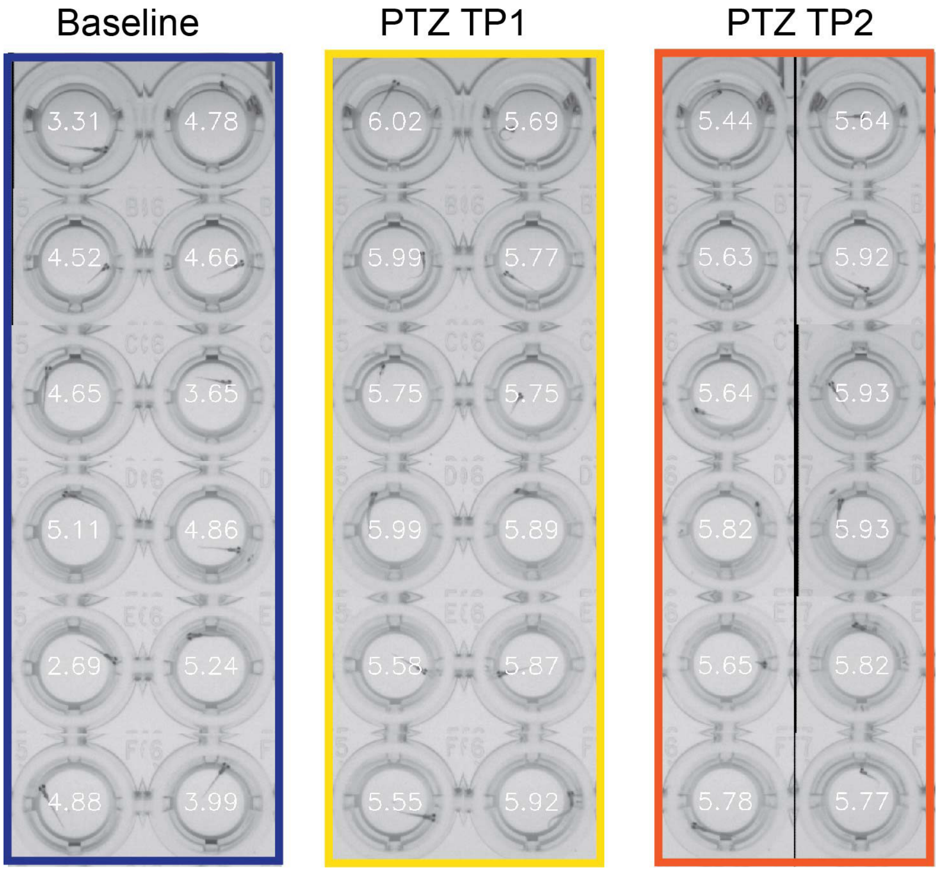

Supplemental Figure 3: Activity Metric. Total activity metric values (∆pixel) on a $\log_{10}$ scale corresponding to tracking plots shown in Figure 1b. Representative examples (12 each) are shown for baseline (blue), PTZ TP1 (yellow) and PTZ TP2 (red).

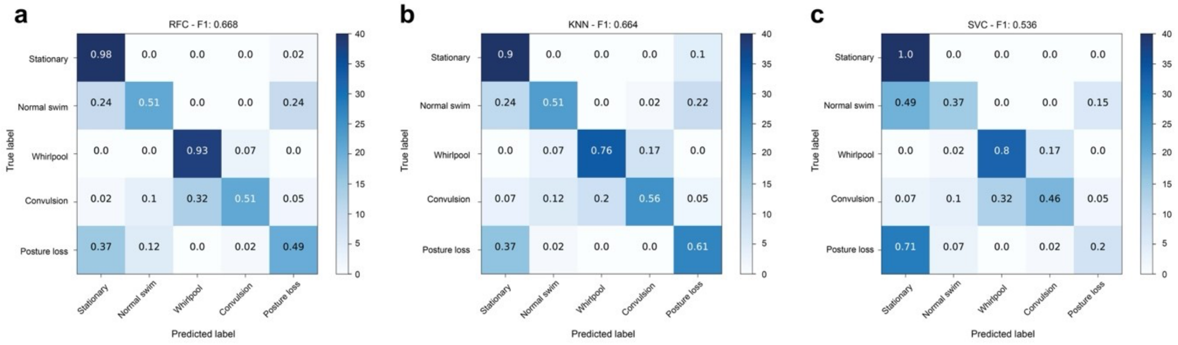

d

$$\text{Precision} = \frac{TP}{TP + FP}$$

$$\text{Recall} = \frac{TP}{TP + FN}$$

e

Algorithm	Random Forest	K-Nearest Neighbors	Support Vector Classifier
**Precision**	0.712	0.688	0.605
**Recall**	0.684	0.668	0.566
**F1**	0.668	0.664	0.536

Supplementary Figure 4: Models. Confusion matrices depicting accuracy of all models tested for behavior classification using a **a.** random forest classifier, **b.** K-nearest neighbor and a **c.** Support vector machine classifier. **d.** Each model was tested for precision and recall scores according to these equations. **e.** Table showing evaluations of all models. These models did not perform well enough and were not utilized in the final behavior classification. The RFC was improved for analysis by increasing training duration and utilizing a different training and test sets.

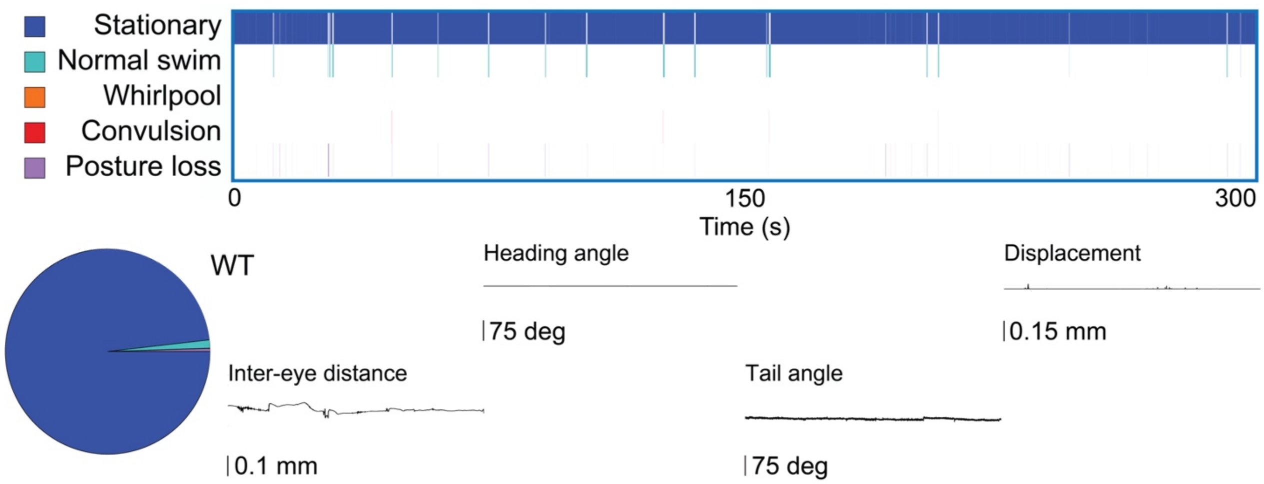

Supplementary Figure 5: WT control. Ethogram plot for a representative WT zebrafish. The entire duration of the 5 min recording epoch for one representative larva is shown (top). Pie chart depicting percentage of each behavior performed at each recording epoch (bottom, left). Color coding as in Fig. 8. Scalar measurements plotted that align with ethogram showing various seizure-like behaviors (bottom, right).
