# Supplementary material for: Automated detection of complex zebrafish seizure behavior at scale
Source: Commun Biol. 2025 Jun 5;8:872. doi: 10.1038/s42003-025-08310-6 (PMC12141442; doi:10.1038/s42003-025-08310-6)
Supplement: Supplementary file 2 — Description of Additional Supplementary Files [file 42003_2025_8310_MOESM2_ESM.pdf]

## Description of Additional Supplementary Files

File name: Supplementary Video 1

Description: Video of the 96 well plate of larvae we capture showing the tracking with the 8 skeletal key points overlaid on each fish

File name: Supplementary Video 2

Description: Sample of the segmented frames showing stationary behavior according the the ML classification algorithm

File name: Supplementary Video 3

Description: Sample of the segmented frames showing normal swim behavior according the the ML classification algorithm

File name: Supplementary Video 4

Description: Sample of the segmented frames showing whirlpooling behavior according the the ML classification algorithm

File name: Supplementary Video 5

Description: Sample of the segmented frames showing posture loss behavior according the the ML classification algorithm

File name: Supplementary Video 6

Description: Sample of the segmented frames showing convulsive behavior according the the ML classification algorithm
